# Experimental Study and Numerical Modeling of Inter-Pass Forging in Wire-Arc Additive Manufacturing of Inconel 718

**DOI:** 10.3390/ma19010182

**Published:** 2026-01-04

**Authors:** Oleg Yu. Smetannikov, Gleb L. Permyakov, Sergey D. Neulybin, Ivan P. Ovchinnikov, Alexander A. Oskolkov, Dmitriy N. Trushnikov

**Affiliations:** Department of Welding Production, Metrology and Technology of Material, Perm National Research Polytechnic University, 29 Komsomolsky Prospect, 614990 Perm, Russia; sou2009@mail.ru (O.Y.S.); gleb.permiakov@gmail.com (G.L.P.); sn-1991@mail.ru (S.D.N.); ivanovchinnikov199@gmail.com (I.P.O.); trdimitr@yandex.ru (D.N.T.)

**Keywords:** WAAM, additive manufacturing, Inconel 718, inter-pass forging, microhardness, residual stress and strain, material properties, mathematical modeling, finite element modeling (FEM), constitutive model of the material, Johnson–Cook law

## Abstract

Inter-pass forging with different degrees of deformation during WAAM of Inconel 718 specimens (single-stage, three passes; two-stage, six passes) was investigated. Macrostructural analysis of the specimens showed that inter-pass forging led to a recrystallized structure. Alternation of layers with different grain shapes (columnar and equiaxed) is observed throughout the height of the specimens. Increasing the number of passes improves the mechanical properties of the material (tensile strength, yield strength, microhardness). A finite element model of inter-pass forging was developed to determine the effect of inter-pass surface deformation during WAAM on the residual stress–strain state. The non-stationary formulation was replaced with a quasi-static one. Johnson–Cook material constants were obtained for the deposited Inconel 718 material, including the effect of forging. Verification of the mathematical model was performed using a wall (specimen 2) deposited with single-stage forging. The deviation between the simulation results and the experiment did not exceed 15%. It was found that the sequence and number of passes significantly affect residual strain and displacements but have little effect on residual stress. Numerical modeling showed that the depth of plastic deformation exceeds the melting depth when depositing the subsequent layer, ensuring the preservation and accumulation of the inter-pass forging effect throughout the deposition process.

## 1. Introduction

Superalloys of the Ni–Cr–Fe alloying system are widely used in the aerospace industry because of their high heat resistance and hot strength [1,2,3]. Such superalloys are used for housings, rings, and sealing elements of structures as well as in oil-field fasteners, valves, and drilling tools [4,5]. However, these alloys are difficult to machine, which makes the development of new approaches to producing billets close in size to the final part topical [6].

Traditionally, parts and structures are manufactured using machining and/or welding of billets produced by casting or wrought performs. Nevertheless, Ni–Cr–Fe superalloys are prone during welding to dendritic liquation and the subsequent formation of the Laves phase in the fusion zone, to micro-cracks in the heat-affected zone due to eutectic phases and carbides along grain boundaries, and to porosity, all of which significantly degrade service properties [7]. Various mechanical and thermal treatments—high-frequency micro-vibrations during welding, ultrasonic processing to prevent cracking, and heat treatment to relieve residual stresses—are used to improve performance [8,9,10]. The resulting chain of welding, mechanical, and heat treatment operations complicates production significantly.

In recent years, alternative methods for producing billets from Ni–Cr–Fe superalloys using hybrid additive (HA) technologies such as Selective Laser Melting (SLM) [11,12], Laser Powder Bed Fusion (L-PBF) [13], Electron Beam Powder Bed Fusion [14], Direct Metal Laser Sintering (DMLS) [15], and Wire-Arc Additive Manufacturing (WAAM) have developed actively [16,17,18,19,20]. WAAM offers high productivity and relatively inexpensive equipment. HA technologies allow the production of billets close in size to the final part to be topical and with high service characteristics. However, these technologies are associated with undesirable phenomena such as non-uniform structure, porosity, residual stresses, and susceptibility to hot cracking [21]. A further challenge is the epitaxial growth of elongated columnar grains throughout the height of the deposit [16,22], causing anisotropy and reduced strength and fatigue properties [23]. Optimizing the deposition strategy or thermal cycles does not fully eliminate these deficiencies [16,24].

Improving the quality of billets produced by HA technologies is possible by combining several techniques, namely inter-pass deformation such as rolling or forging during deposition and subsequent heat treatment [16,19,20,25].

In works [26,27], the influence of inter-pass rolling during the three-dimensional deposition on the formation of billets from the titanium alloy Ti-6Al-4V is investigated. It is shown that rolling leads to a decrease in substrate deformations from 7 to 3 mm, and a refinement of the structure: primary β grains decreased from 30 mm to 89 μm, the length of the α phase plates decreased from 21 μm to 8 microns, and the width of the α phase plates decreased from 1.2 μm to 0.7 μm. Rolling also led to an increase in mechanical properties: tensile strength of 1078 MPa, relative elongation of 14%. Similar results were demonstrated during the inter-pass rolling of specimens from low-carbon structural steel [28]. Moreover, the higher the rolling load is, the more the structure of the deposited metal is refined. It is also shown that the refinement of the structure of the rolled layer occurs after heating during the deposition of the subsequent layer. During the three-dimensional deposition of simple linear-shaped parts, rolling is a highly applicable and effective deformation method. However, the practical application of this method for the three-dimensional deposition of more complex parts using inter-pass rolling can present some challenges. Deformation of complex-shaped parts in arbitrary directions within a layer is difficult. The rolling force is significant (70–10 kN), making the equipment complex and expensive. Furthermore, such high loads can easily cause irreparable deformation or destruction of the deposited parts. Therefore, inter-pass forging should also be considered as a more flexible and versatile deformation method in terms of the variety of billet geometries.

The author of [29] proposed using inter-pass forging with a pneumatic impact tool to improve the quality of deposited steel billets. Research [30,31] explores the potential of mechanical impact treatment, which can complement or replace rolling as a method of inter-pass cold treatment, since mechanical impact treatment tools can be easily manipulated with a greater degree of freedom. It has been shown that the refinement of β grains of the deposited titanium alloy occurs in the heat-affected zone (β region) during the deposition of the subsequent layer. Based on the results of these studies [32,33], scientists conclude that the disadvantages of wire-arc additive technologies, such as porosity and unfavorable structure, can be compensated for by mechanical impact treatment. Mechanical impact treatment allows for material compaction, refinement of the structure, and the improvement of mechanical properties.

Research has also revealed that the billet material influences the effectiveness of mechanical impact treatment, requiring that preferred modes be selected for each material to achieve the best results. The shapes of the tool (hammer) and waveguide have the greatest impact on treatment efficiency. Regarding the geometric parameters of the billets, interesting results show that the greatest mechanical impact treatment efficiency is achieved when the width of the billet is equal to or less than the diameter of the hammer.

In the study [34], inter-pass forging strengthening during the deposition of the Ti-6Al-4V titanium alloy reduced the primary β-grain size in the *z*-axis direction, i.e., in the deposition direction, from an average size of 120 mm to an average size of 460 μm. Inter-pass forging also led to an increase in the number of α-colony nucleation sites and reduced the length and width of the α-grains, as with inter-pass rolling. The width of the α grains without forging is 1.4 μm, and with forging is 0.8 μm. The length of the α grains without forging is 25.5 μm, and with forging is 14.8 μm. Inter-pass forging leads to an increase in the yield strength and tensile strength without reducing elongation, and leads to a decrease in the anisotropy of the material. Unfortunately, the main text of the article is written in Korean, so it is difficult to provide any additional information beyond the main conclusions in terms of analyzing the mechanisms of structure refinement and property improvement.

This paper [35] examines the effect of inter-pass deformation treatment and subsequent general heat treatment on the quality of deposited metal in three-dimensional wire-arc deposition. Subsequent general heat treatment of unforged specimens does not significantly improve the mechanical properties of the deposited metal. The use of inter-pass forging during wire-arc deposition improves the structure of the deposited metal and increases its strength (1050–1060 MPa). The subsequent general heat treatment of forged specimens leads to an additional refinement of the macro- and microstructure, improving ductility (relative elongation up to 13–14%) while maintaining the high strength of the deposited metal (980–1000 MPa). The specimens produced by three-dimensional deposition with controlled heat input, inter-pass deformation treatment, and subsequent heat treatment demonstrate high quality, characterized by the stability of geometric characteristics, the almost complete absence of anisotropy, and a guaranteed level of mechanical properties for the material of forgings made of Ti-6Al-4V alloy (OST 1 90000-70).

Mathematical modeling can be used to determine the behavior of metal and to determine the parameters and effectiveness of inter-pass surface deformation, in particular the depth of plastic deformation and the degree of distortion of the produced part.

In [36], the influence of various rolling process parameters, in particular the rolling load and the roller profile radius on the distribution of plastic strain, was investigated using the finite element method. The influence of roller hardness was investigated numerically. The results show that, under the same rolling load, a deformable roller produces lower equivalent plastic strains due to its own elastic deformation. In addition, a lower friction coefficient causes higher equivalent plastic strains near the rolled surface, but has little effect on the plastic strain at a greater depth. However, with a higher friction coefficient, the calculation time increased significantly. Larger roller profile radii lead to a decrease in plastic strain near the rolled surface, but at the same time have almost no noticeable effect on the plastic strain at a greater depth.

In addition, the influence of the gap between the rollers on the uniformity of plastic deformation during multi-pass rolling was investigated using a selected example. The calculation results show that a more uniform distribution of plastic deformation is achieved when the distance between two rollers is equal to the residual groove width obtained in one rolling pass. The study [37] uses finite element modeling of the rolling process to investigate the influence of rolling parameters, in particular the rolling load and the roller profile radius, on the residual stress field, as well as on the distribution of plastic deformation.

Results are shown for structural metals commonly used in 3D deposition: AA2319 and S335JR steels, and Ti-6Al-4V titanium alloy, considering the presence of residual stresses. The rolling load changes the position and maximum value of compressive residual stresses, as well as the depth of these compressive residual stresses. However, the roller profile radius changes only the maximum value of these compressive residual stresses. A change in the rolling load affects the equivalent plastic strain near the rolled surface, as well as in deeper sections, while the influence of the roller profile radius can be neglected. The distribution of plastic strain is practically unaffected by the initial residual stresses before rolling.

Reference [38] proposed a numerical model to assess how surface processing of the deposited parts affects their residual stress–strain state (RSS). The simulations were performed in LS-DYNA^®^ R13 using a quasi-static finite element formulation accounting for geometric and elastoplastic nonlinearities. The authors experimentally studied the effects of geometry, alloy type, and processing depth on hardness and residual stresses. Using LS-DYNA^®^ to simulate the entire deposition process is problematic because it lacks element “kill/revive” functionality (Ekill/Ealive) used to simulate the natural stress state of a newly deposited layer. This study attempts to assess the applicability of classical implicit solvers in ANSYS 2021 Mechanical APDL to problems of inter-pass surface deformation during the additive manufacturing of structures.

## 2. Materials and Methods

To produce experimental specimens, Inconel 718 welding wire was used; its chemical composition is given in Table 1.

**Figure 1:**
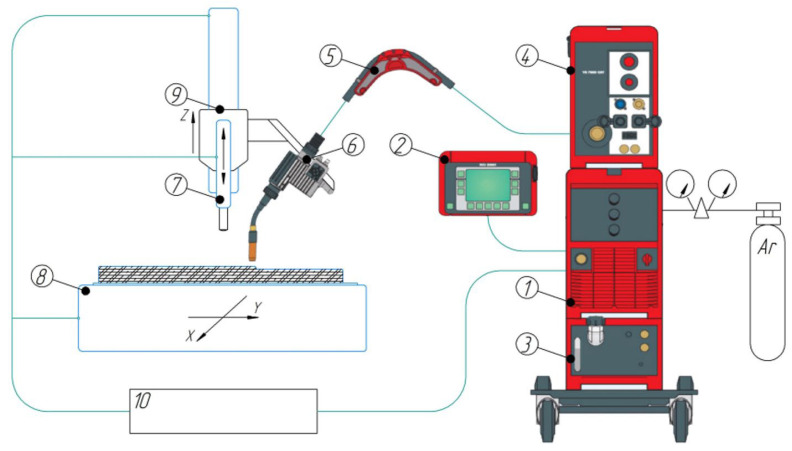
Functional scheme of the hybrid CMT-WAAM forging setup: (1) welding power source; (2) remote control setup; (3) chiller FK4000-R; (4) wire feeder; (5) wire buffer CMT; (6) welding torch; (7) pneumatic hammer; (8) two-axis table; (9) machining-center column; (10) control panel.

**Figure 2:**
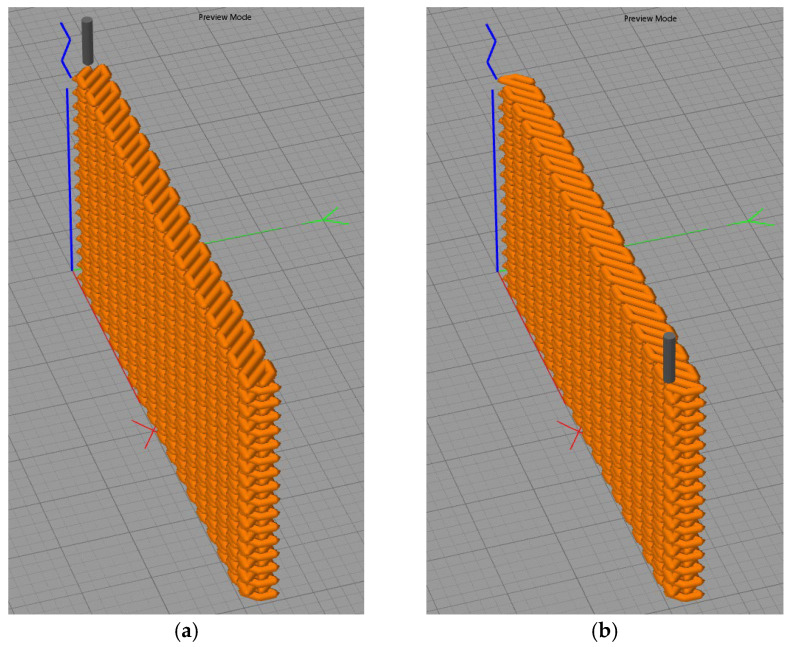
The schemes of the deposition strategy with (**a**) 45° and (**b**) −45° oscillations.

Wire-arc deposition was carried out on a hybrid manufacturing setup (Figure 1) assembled on a standard 3-axis vertical machining center chassis (AT-300, Hybrid Additive Manufacturing Group of Companies, McKinney, TX, USA). The setup was equipped with interfaces to a welding power source and an Evospark torch, and was controlled by an integrated CNC. The Evospark 500 source (Evospark, Lippstadt, Germany) provided digital process control. Cold metal transfer with short-circuiting was employed with the following parameters: arc current I = 140–170 A; arc voltage U = 13–15 V; wire feed rate V_f_ = 6.0–7.0 m/min; torch travel speed V_d_ = 40–50 cm/min; argon shielding gas flow Q_s_ = 25 L/min. To study the effect of deformation intensity on structure and properties of specimens, the degree of deformation was controlled by processing deposited layers with a pneumatic hammer. The forging parameters were as follows: hammer travel speed V = 300 mm·min^−1^; stroke frequency N = 2820 strokes·min^−1^; impact energy E = 19.74 J; tool shape—a hemisphere of radius R = 15 mm; and contact force 300 N. To ensure uniform deformation, forging was performed over the entire surface of each deposited layer. In the first case, a single-stage forging was used; in the second case, two-stage forging, i.e., double forging of the deposited layer. Each forging stage comprised three longitudinal parallel passes (single-stage forging in three passes, two-stage forging in six passes over the surface of the specimen). Due to different cooling times, the inter-pass surface temperature during forging was ~300 °C for single-stage and ~200 °C for two-stage processing. As a result, wall specimens were obtained (length × width × height 200 × 50 × 18 mm). Multi-layer deposition followed a ±45° oscillating path. The layer sequence is shown in Figure 2. Three specimens were produced: without pneumatic forging (specimen 1); with single-stage forging (specimen 2); and with two-stage forging (specimen 3). Metallography was performed on cross-sections of the walls. Macrostructure was examined on an Altami CM0745-T stereomicroscope (Altami, St. Petersburg, Russia) using Altami Studio 4.0. A Vasiliev reagent (CuSO_4_—20 mL, H_2_SO_4_—75 mL, HNO_3_—5 mL) was used to reveal macro- and microstructure. Microhardness was measured by the Vickers method at a load of 100 g (HV_0.1_) using an EMCO-TEST DuraScan-70 g5 tester (Emco-test, Kuchl, Austria). Measurements were made in the central part of specimens over an area 3 × 6 mm with 0.2 mm spacing; 450 measurements were performed on each specimen.

**Figure 3:**
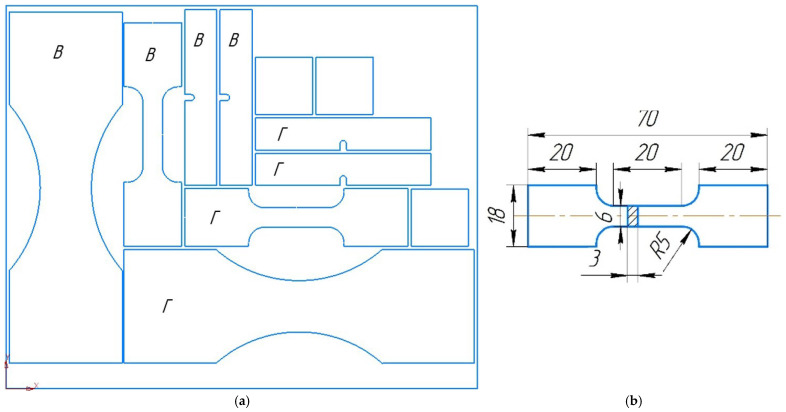
A scheme of specimen cutting and sketches of specimens (**a**) and a sketch of the static tensile specimen (**b**).

The measurement area was selected in the center of the deposited billet. The measurement area exceeds the height of one layer. Therefore, the measurements include both the layer itself and the interlayer region, allowing for an assessment of both the effect of additional strain hardening and possible softening from subsequent deposition layers. The measurement is performed automatically, and therefore no special selection of the measurement location was required. To assess general mechanical properties, tensile specimens were cut from the walls (Figure 3a). Specimens were cut from the deposited billets according to a scheme of specimen cutting. A sketch of the static tensile specimen is shown in Figure 3b. Five specimens of each type were cut from the wall cross-section for mechanical testing, including anisotropy assessment in the horizontal and vertical directions.

Flat specimens for static tension were tested on an Instron 8802 machine (Instron, Norwood, MA, USA) at a strain rate of 1 × 10^−3^ s^−1^.

Study [39] has shown that light-colored stringy inclusions of both cubic and irregular shapes were detected in the microstructure of the deposited material. In the former zone, the distance between these particles is 20–100 μm, which coincides with the average dendrite thickness. The particle thickness is 10.0 ± 8.2 μm. The irregularly shaped particles are the Laves phase. These Fe-Cr-Nb particles have an elevated Nb content. Apparently, the Laves phase formed during cooling according to the mechanism L → γ → γ + MC → γ + MC + Laves phase. Additionally, cubic inclusions measuring 0.6 ± 0.3 μm are observed in the structure. These particles have a high Ti and Nb content and were identified as (Nb, Ti)(N,C) carbides. Furthermore, particles detected with matching maximum ROI intensities in the EDAX maps for Ti and N (red dotted line) were identified as TiN nitrides. In the second zone of the deposited metal, Laves phase particles are significantly smaller in size—4 ± 1.8 µm, due, on the one hand, to fragmentation during forging and, on the other, to partial dissolution under heating during deposition of the subsequent billet layer.

The same article examined the mechanical properties of the material and their failure characteristics, including those observed during subsequent heat treatment. Analysis of mechanical test results for the deposited material, both with and without strain hardening, as well as before and after heat treatment, revealed that heat treatment promoted the precipitation of γ′ and γ″ strengthening phases in the FCC matrix, resulting in an increase in strength by approximately 45%. Viscous fracture is the primary failure mechanism for specimens under tension. At lower magnification, differences in the dimple structure between specimens before and after heat treatment were observed. Thus, specimens of deposited material before heat treatment fracture along the interlayer boundary running between the forged zones.

Microcracks were detected in the brittle intermetallic Laves phase at the fracture site. The deposited material after heat treatment fractures primarily along the boundaries of coarse grains, while microcracks were found primarily along the boundaries and cores of carbonitride particles. The impact toughness of transversely oriented specimens was 65 ± 10 J/cm^2^, due to the additional energy required to inhibit cracks upon encountering fine grain boundaries.

A comparison of the microstructural study and fractographic analysis results suggests that the initial Laves phase particles facilitate crack initiation and propagation, as was also observed under uniaxial tension. Clearly, the increase in ductility and impact toughness, while maintaining the coarse-grained structure after heat treatment, is due to the dissolution of Laves phase particles, which, due to their irregular shape, act as stress concentrators and thus promote early crack formation and fracture. In turn, the enrichment of the solid solution with niobium and titanium due to Laves phase dissolution ensured the precipitation of γ′ and γ″ phase particles in the matrix, significantly strengthening the deposited material.

Constitutive model of the material.

Large plastic strains and small elastic strains in metals were described using the standard Johnson–Cook (J–C) constitutive law [40,41] written in an updated Lagrangian formulation expressed in terms of the stress rate and rate of strain tensors. Additivity of elastic and plastic strain rate tensors is assumed. The elastic tensor is linearly related to the Jaumann derivative of the Kirchhoff stress tensor, and the plastic strain rate tensor is related to the Cauchy stress tensor by the associated flow rule:

(1) $${\dot{\varepsilon }}_{ij}^{p}=\dot{\lambda }\frac{\partial g}{{\partial \sigma }_{ij}},$$

where ${\dot{\varepsilon }}_{ij}^{p}$ are components of the plastic strain rate tensor, ${g(\sigma }_{ij})$ is the plastic potential (taken as the yield function), $\sigma_{ij}$ are components of the stress tensor, and $\dot{\lambda }$ is a plastic multiplier (Lagrange multiplier) that determines the magnitude of plastic deformation. The plastic potential associated with the von Mises yield criterion is assumed:

(2) $${g(\sigma }_{ij})=\sigma_{u}=\sigma_{i},$$

where $\sigma_{i}=\sqrt{3s_{ij}s_{ij}/2}$ is the equivalent stress, $s_{ij}=\sigma_{ij}-\sigma_{m}\delta_{ij}$ are components of the stress deviator, $\sigma_{m}=\sigma_{kk}/3$ is the mean stress, $\delta_{ij}$ is the Kronecker delta, and $\sigma_{u}$ is the yield strength in uniaxial tension described by the Johnson–Cook isotropic hardening law [42,43]:

(3) $$\sigma_{u}=\left[A+B\varepsilon_{p}^{n}\right]\left[1+C\ln\frac{{\dot{\varepsilon }}_{p}}{{\dot{\varepsilon }}_{*}}\right]{\left[1-\frac{T-T_{amb}}{T_{liq}-T_{amb}}\right]}^{m},$$

where ${\dot{\varepsilon }}_{p}=\sqrt{2{\dot{\varepsilon }}_{ij}^{p}{\dot{\varepsilon }}_{ij}^{p}/3}$ is the equivalent plastic strain rate; $\varepsilon_{p}=\int_{0}^{t}{\dot{\varepsilon }}_{p}dt$ is the equivalent plastic strain; A,B,C,n,m are material constants; ${\dot{\varepsilon }}_{*}$ is a reference strain rate; $T_{liq}$ = 1609 °C is the liquidus temperature; $T_{amb}$ is ambient temperature; T is absolute temperature. The plastic multiplier $\dot{\lambda }$ can be found as follows:

(4) $$\dot{\lambda }=\frac{3{\dot{\varepsilon }}_{p}}{{2\sigma }_{i}},$$

In works [44,45], the adequacy of replacing the non-stationary formulation with a quasi-static one without significant loss of accuracy was proven by calculation using the example of an axisymmetric problem of the impact of a hemispherical hammer with a plastic cylinder. The pass of the impact tool was replaced by the pass of a roller compactor, and it was shown that with appropriate calibration the resulting distributions of the stress–strain state parameters are identical.

For alloys AMg6, 12Cr18N10T, and Ti-6Al-4V, the impact was calibrated in a numerical model of forging with a pneumatic hammer and the equivalent depth of rolling tool indentation was determined when modeling roller compaction.

That is, the indentation depth of the roller tool that produces a result equivalent to the forging was used. For AMg6, 12Cr18N10T, and Ti-6Al-4V materials, the equivalent indentation depths were 1.2 mm, 0.6 mm, and 0.3 mm, respectively. The equivalent calculated tool indentation force was ~44,000 N. This result demonstrates the advantages of forging over rolling. A load of 44,000 N, equivalent to forging, requires complex, expensive rolling equipment and will result in the collapse of insufficiently rigid structures. When subjected to impact, the equipment presses the tool with a force two orders of magnitude lower, ~300N. Impact forces are also not significant, as they are initially intended for manual use. The experimental part [44,45] of the equipment used for forging and the forging modes are identical to those presented in the present work. Similarly, the methodology described in [44,45] is applied in this work for the Inconel 718 alloy. A vertical load of $F_{y}=44,000\;N$ is applied to the equivalent rolling tool nodes, uniformly distributed between them.

The yield strength of Inconel 718 alloy in the as-deposited condition (without subsequent heat treatment) is slightly higher than that of 12Cr18N10T steel. Therefore, the equivalent tool indentation depth, as calculated, will be slightly lower: up to 0.3 mm.

The possibility of replacing the non-stationary formulation with a quasi-static one is substantiated in [44]. This substantiation is provided at two levels:

- By comparing the dynamic (implicit) and quasi-static solutions to the problem of a high-speed collision of a massive sphere with an elastic–plastic cylinder, where the insignificance of the influence of inertial terms is demonstrated.
- By comparing the solutions to the specimen forging problem in a dynamic (explicit, DYNA) formulation, performed by other authors and by us in a quasi-static formulation (ANSYS).

A feature of the physical model used in this work (as well as in [44]) is the adaptation of the Johnson–Cook model, which is absent in ANSYS 2021 Mechanical, to the multi-isotropic nonisothermal plasticity model MISO. It is assumed that the average strain rate $\left\langle {\dot{\varepsilon }}_{pr}\right\rangle$ is constant and specified for a given forging process. The calculation of $\left\langle {\dot{\varepsilon }}_{pr}\right\rangle$ is performed based on preliminary data on the dynamics of the working tool (indenter). A numerical experiment is conducted on the collision of a rigid massive sphere of radius $R_{pr}$ with an elastic–plastic half-space, while its kinetic energy $E_{k}$ must correspond to the collision energy of the tool used for forging.

(5) $$\left\langle {\dot{\varepsilon }}_{pr}\right\rangle =\varepsilon_{max}(V_{pr})/∆t_{ind},$$

Here, $\varepsilon_{max}$ is the maximum equivalent strain in the dimple at the moment the sphere begins to rebound, $V_{pr}=\sqrt{E_{k}/M_{pr}}$ is the initial velocity of the sphere, $M_{pr}$ is the mass of the indenter, and $\Delta t_{ind}$ is the time interval from contact with the indenter to the onset of rebound.

The resulting value of $\left\langle {\dot{\varepsilon }}_{pr}\right\rangle$ is substituted into (3) for the subsequent transition to the MISO model. The value of $\left\langle {\dot{\varepsilon }}_{pr}\right\rangle$ found at room temperature is also used for other temperatures $T_{i},i=1\ldots N_{T}$ in the operating range, which are substituted in (3) one by one.

From the above, it follows, in particular, that if the forging mode changes significantly, the adaptation procedure in MISO should be repeated. The use of the described method is supported by the fact that for most materials, an order-of-magnitude change in strain rate changes the yield strength by 1–2%.

A partial description of the model identification process is provided in [45]. The MISO model is a table of pairs of values $\varepsilon_{i}^{k},\;\sigma_{i}^{k}$, where $i\in [1,\;N_{pt}^{K}]$, $k\in [1,N_{T}]$, $N_{T}$ is the number of fixed temperatures in the process interval, and $N_{pt}^{K}$ is the number of points on the uniaxial loading diagram for the k-th temperature. The first pair determines the initial yield strength. The $\varepsilon_{i}^{k},\;\sigma_{i}^{k}$ table, constructed using Formula (3) in MathLab R2017b for a given temperature and average strain rate, is transferred to ANSYS 2021 via text files.

## 3. Results and Discussion

### 3.1. Effect of Inter-Pass Forging on the Structure of the Material

Tests were conducted on specimens produced by arc deposition with ±45° oscillation:

- Without inter-pass forging (specimen 1);
- With single-stage forging in three passes (specimen 2);
- With two-stage forging in six passes (specimen 3).

**Figure 4:**
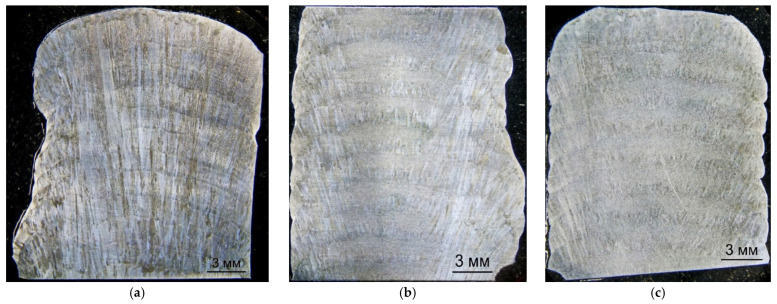
Macrostructure of the deposited wall in cross-section, magnification ×4: (**a**) deposition without forging; (**b**) deposition with single-stage forging; (**c**) deposition with two-stage forging.

In the macrostructure of specimen 1, long columnar grains oriented vertically and extending through almost all deposited layers were observed. Trans-crystalline growth of the columnar grains occurs opposite to the direction of maximum heat extraction. The oscillating strategy produced layers about 2 mm in height. At interlayer boundaries, the fraction of regions retaining the preceding crystallographic orientation decreases. Areas appeared where dendrites of the new layer were oriented differently relative to dendrites of the previous layer (Figure 4).

Inter-pass cold forging, irrespective of the number of passes, resulted in regions with fine equiaxed grains. Alternating layers of columnar and equiaxed grains were observed (Figure 4). With single-stage forging, this alternation was noticeable mainly in the central part of the specimen; at the edges, mainly columnar grains were observed, whose length was limited to the height of one layer—probably due to non-uniform deformation across the wall width. Two-stage forging increased the volume of equiaxed grain regions and significantly reduced the fraction of columnar grains. Alternating layers extended across the full width of the specimen. Table 2 presents the geometrical measurements of the layers with different grain shapes.

Macrostructural analysis showed that inter-pass forging led to a recrystallized structure due to thermal exposure during the deposition of the subsequent layer (Figure 4b,c). A greater number of forging passes promoted a finer recrystallized structure. In specimen 2 (single-stage), recrystallization occurs primarily near the tops of the deposited layers; in specimen 3 (two-stage), recrystallisation extended through the full height of the deposited layer.

**Figure 5:**
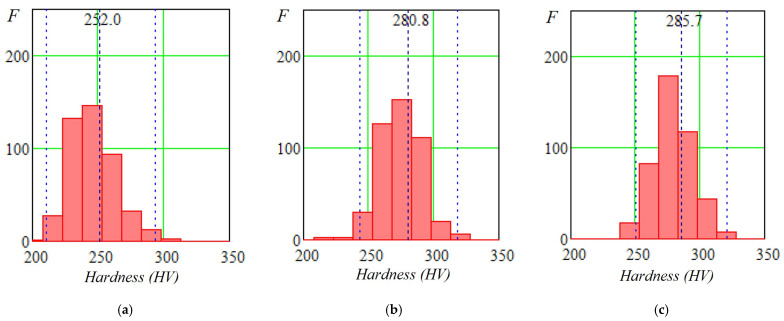
Histograms of HV_0.1_ microhardness distribution: (**a**) deposition without forging; (**b**) single-stage forging; (**c**) two-stage forging; F—frequency (number of values) in the interval.

**Figure 6:**
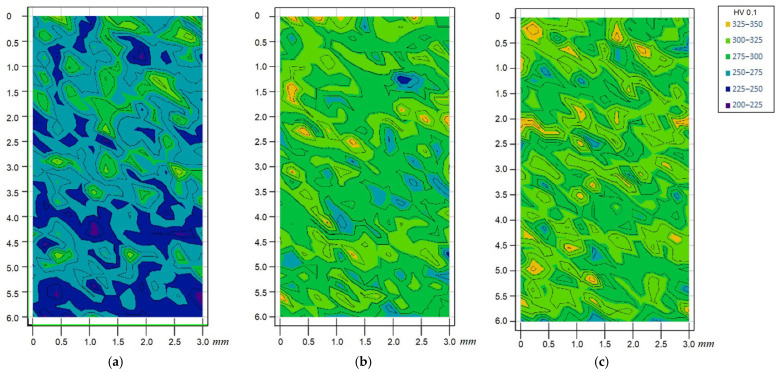
Map of HV_0.1_ microhardness distribution over a 3 × 6 mm area in the specimen center: (**a**) deposition without forging; (**b**) single-stage forging; (**c**) two-stage forging.

The purpose of microhardness measurements was to evaluate the uniformity of the emerging structure and the degree of heterogeneity of mechanical properties at the micro level. Microhardness maps were constructed over a 3 × 6 mm area with a 0.2 mm step, and statistical analysis was performed. Results are given in Table 3 and Figure 5; microhardness maps are shown in Figure 6.

Analysis showed that two-stage forging increased the general microhardness level and produced a more uniform distribution relative to single-stage forging. The range of microhardness values decreased due to an increase in the minimum values. Compared with deposition without inter-pass deformation, forging increased average microhardness by about 20–30 HV_0.1_.

Thus, increasing the number of passes during inter-pass cold forging in the WAAM of Inconel 718 increased the degree of deformation and the depth of plastic deformation. Two-stage forging produced a recrystallized structure over almost the entire height and width of the deposited layer.

### 3.2. Effect on Mechanical Properties and Johnson–Cook Parameters

The constants $A,B,C,n,m,{\dot{\varepsilon }}_{*}$ of the Johnson–Cook hardening law for Inconel 718 material were taken from [46] and adapted in accordance with the results of the uniaxial tensile testing of the deposited specimens, considering single- and two-stage forging (Table 4) without subsequent heat treatment. *E* is the Young’s modulus;$\;\sigma_{B}$ is the tensile strength; $\sigma_{pl}$ is the proportionality limit; δ is the relative elongation; ψ is the relative narrowing.

Table 5 provides Johnson–Cook constants from [46] (after heat treatment) and adapted values for as-deposited and two-stage forged materials. Adaptation was necessary because [46] reports properties after strengthening heat treatment. From Table 5, it follows that its yield strength (constant A) is 1290 MPa, while the values observed in the experiment are in the range of 462–624 MPa. To determine adapted constants, the Johnson–Cook law (Equation (3)) was implemented in MATLAB R2017b and diagrams σ−ε were plotted at room temperature at the experimental strain rate; A and B were fitted to achieve the best agreement in $\sigma_{B}$, $\sigma_{u}$, δ.

Next, the observed elastic–plastic behavior was implemented in ANSYS 2021 as a multi-linear isotropic plasticity model.

### 3.3. Finite Element Modeling of Inter-Pass Forging

**Figure 7:**
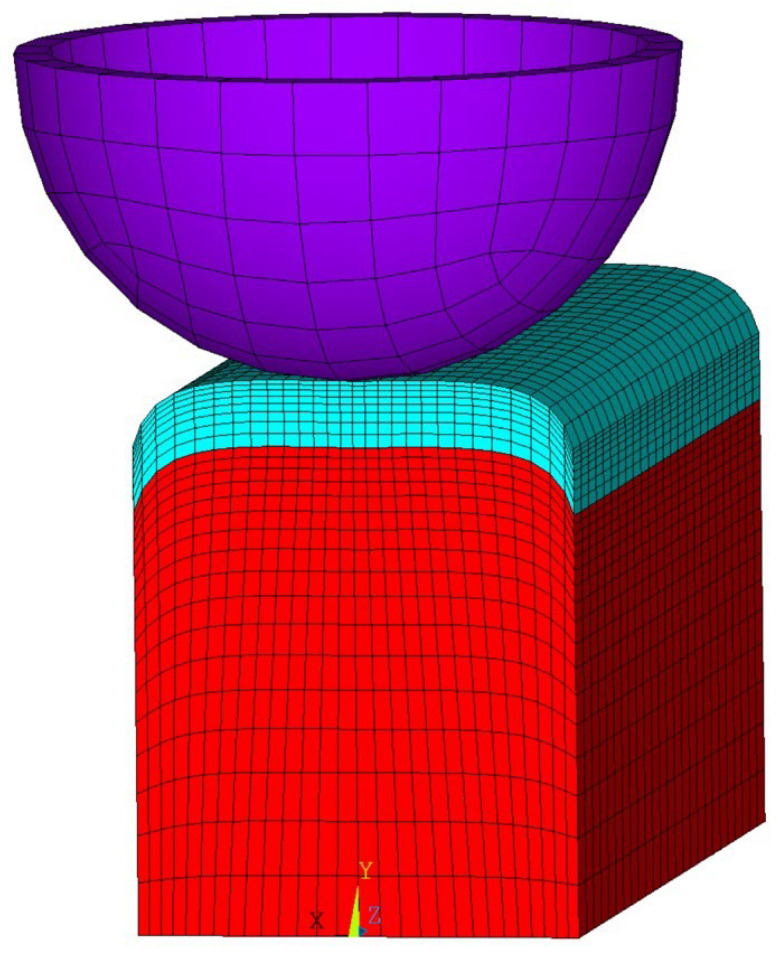
Finite element model considering strengthening of base (previously deposited) layers due to forging.

A 3D finite element model (Figure 7) of the inter-pass forging of the deposited layer was built in ANSYS 2021 Mechanical APDL. The specimen dimensions were a = 20 mm, b = 25 mm, l = 50 mm; the rolling tool radius was R_bit_ = 14.4 mm. The elements used were Solid185 (specimen), Solid186 (rolling tool), Targe170, and Conta174. Boundary conditions were as follows: at the rear boundary of the specimen, the displacements are ${\left.U_{z}\right|}_{z=0}=0$, at the lower boundary ${\left.U_{x,y,z}\right|}_{y=0}=0$. On the rolling tool, depending on the forging program, the displacements in the xz plane are set to ${\left.U_{x,y,z}\right|}_{y=0}=0$. A uniformly distributed vertical load of $F_{y}=44,000H$ was applied to the rolling tool nodes. The rolling tool passes were arranged symmetrically at 3 mm spacing, with pass length $L_{tr}=$ 40 mm.

The specimen volume was divided into two parts: a previously deposited base volume (red in Figure 7) and a newly deposited layer 2 mm height (turquoise).

Figure 8, Figure 9, Figure 10, Figure 11, Figure 12, Figure 13, Figure 14, Figure 15 and Figure 16 shows the results for two calculation cases that differ in the order of the rolling tool passes:

- Case 1 (PC1): Left ↑, central ↓, right ↑; left ↓, central ↑, right ↓.
- Case 2 (PC2): Central ↑, left ↓, right ↑; central ↓, left ↑, right ↓.

Analysis (Figure 9, Figure 12, Figure 13 and Figure 16) showed that successive passes significantly shift material into regions affected by neighboring passes. The distribution of vertical residual displacements in Case 1 (PC1) after the second pass (Figure 12b) is similar to the first pass (Figure 12a); however, the absolute maxima increase by about 1.5 times. Case 2 (PC2) shows a smaller (≈12%) difference between the first (Figure 16a) and second (Figure 16b) passes; the non-uniformity of the residual displacements is similar in both cases. The residual displacement non-uniformity is comparable for both sequences.

Residual stresses (Figure 10 and Figure 14) change little upon double-forging; the average increase does not exceed 10%. Residual strains, in contrast, grow more substantially in some sections—for Case 1 (PC1), this occurs in the left and central sections (Figure 11), and for Case 2 (PC2) also in the left and central sections (Figure 15). Residual strains after the second forging stage increase by more than 100% in some sections.

**Figure 8:**
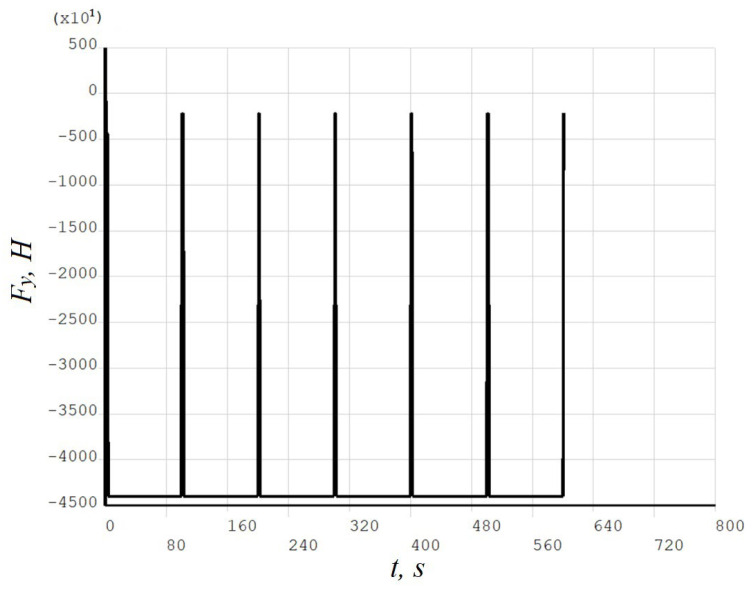
Case 1 (PC1). Time dependence of vertical load $F_{y}$.

- → Case 1 (PC1) Results

**Figure 9:**
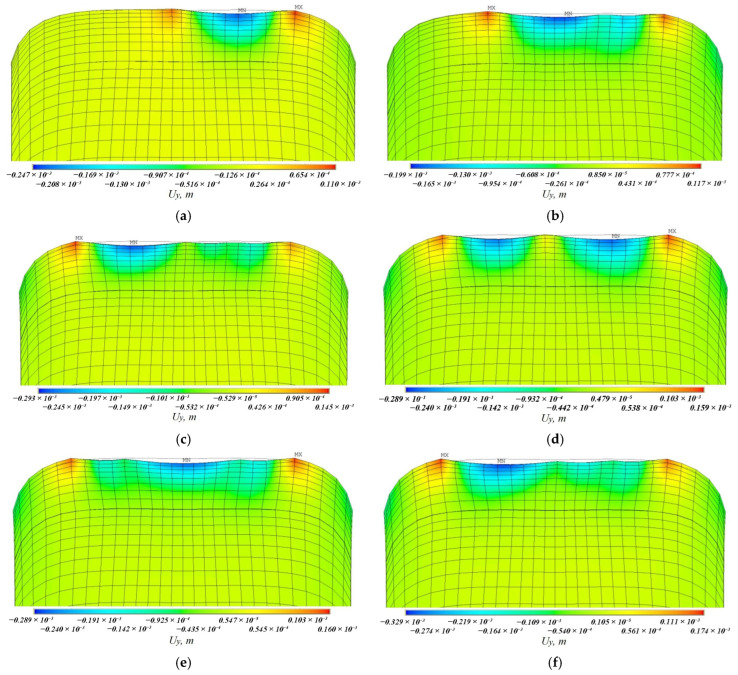
Case 1 (PC1). Vertical residual displacements $u_{y}\;$after: (**a**) the first; (**b**) the second; (**c**) the third; (**d**) the fourth; (**e**) the fifth; (**f**) the sixth passes.

**Figure 10:**
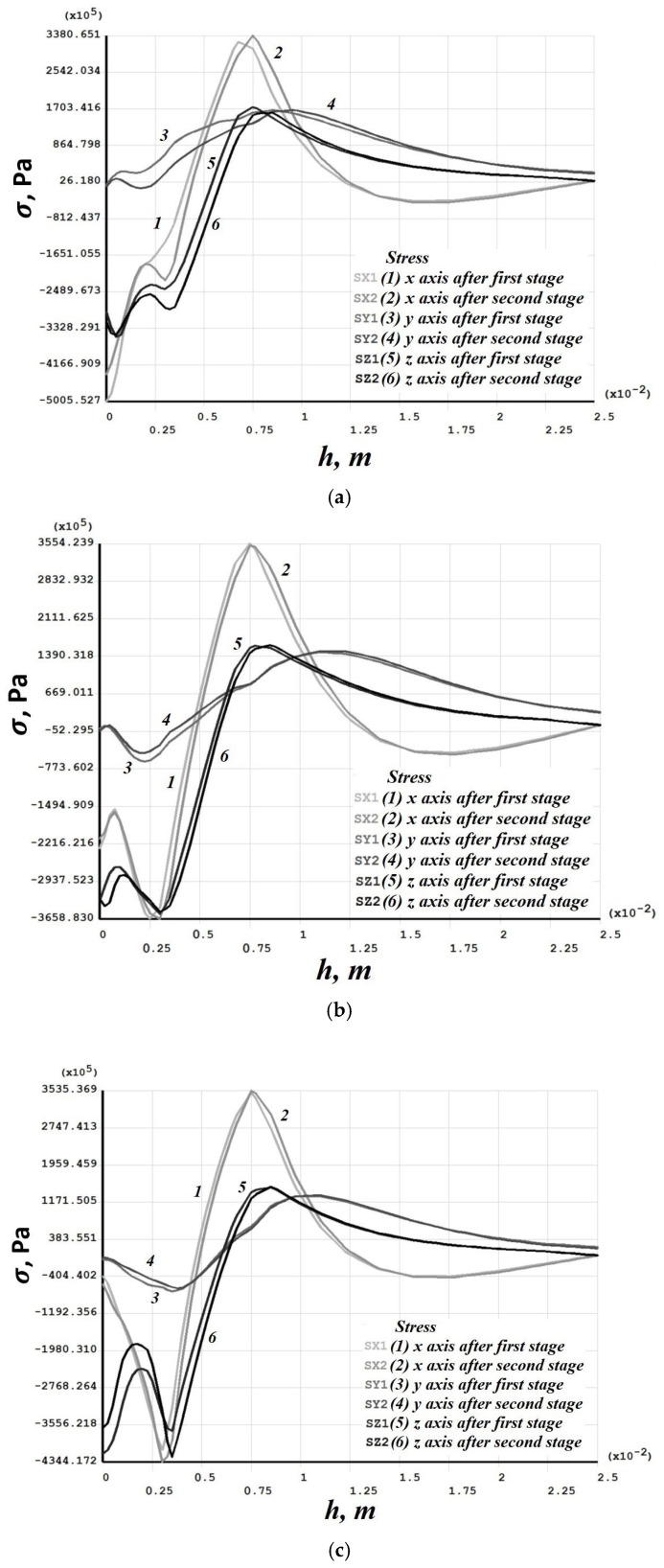
Case 1 (PC1). Distribution through the depth of the residual stress after the first and second stages: (**a**) across the height of the left (−3 mm); (**b**) central (0 mm); (**c**) right (+3 mm) sections.

**Figure 11:**
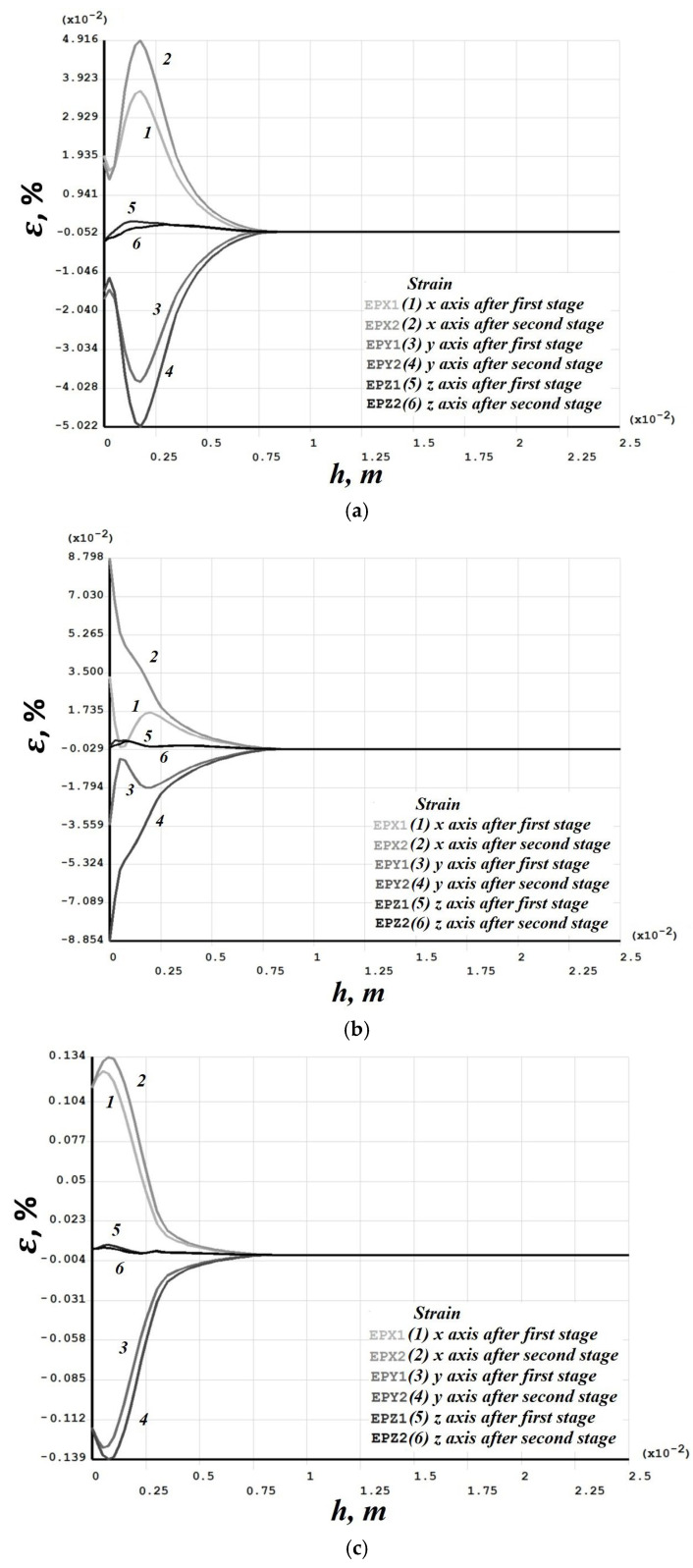
Case 1 (PC1). Distribution through the depth of the residual strain after the first and second stages: (**a**) across the height of the left (−3 mm); (**b**) central (0 mm); (**c**) right (+3 mm) sections.

**Figure 12:**
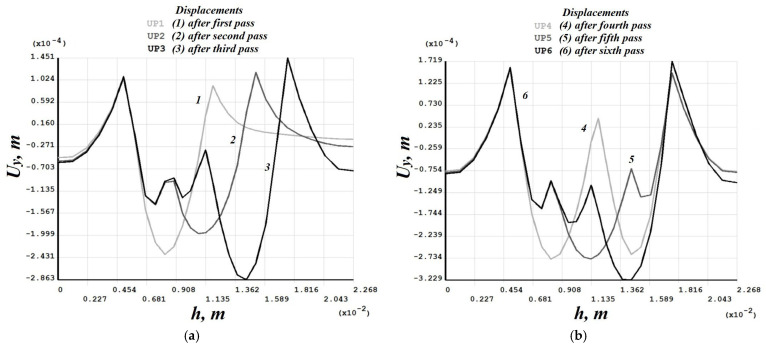
Case 1 (PC1). Distribution of vertical residual displacements along the top surface of the cross-section after the first–third (**a**) and fourth–sixth (**b**) passes.

- → Case 2 (PC2) Results

**Figure 13:**
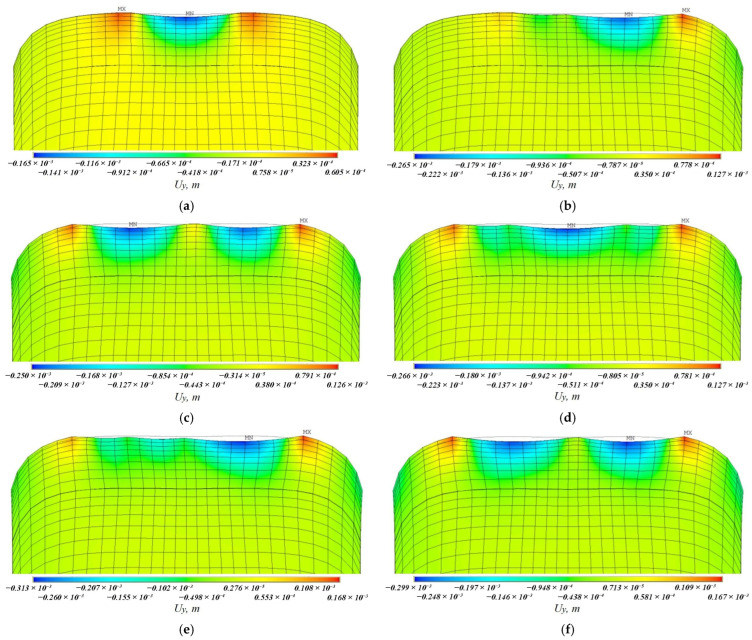
Case 2 (PC2). Vertical residual displacements $u_{y}$ after: (**a**) the first; (**b**) the second; (**c**) the third; (**d**) the fourth; (**e**) the fifth; (**f**) the sixth passes.

**Figure 14:**
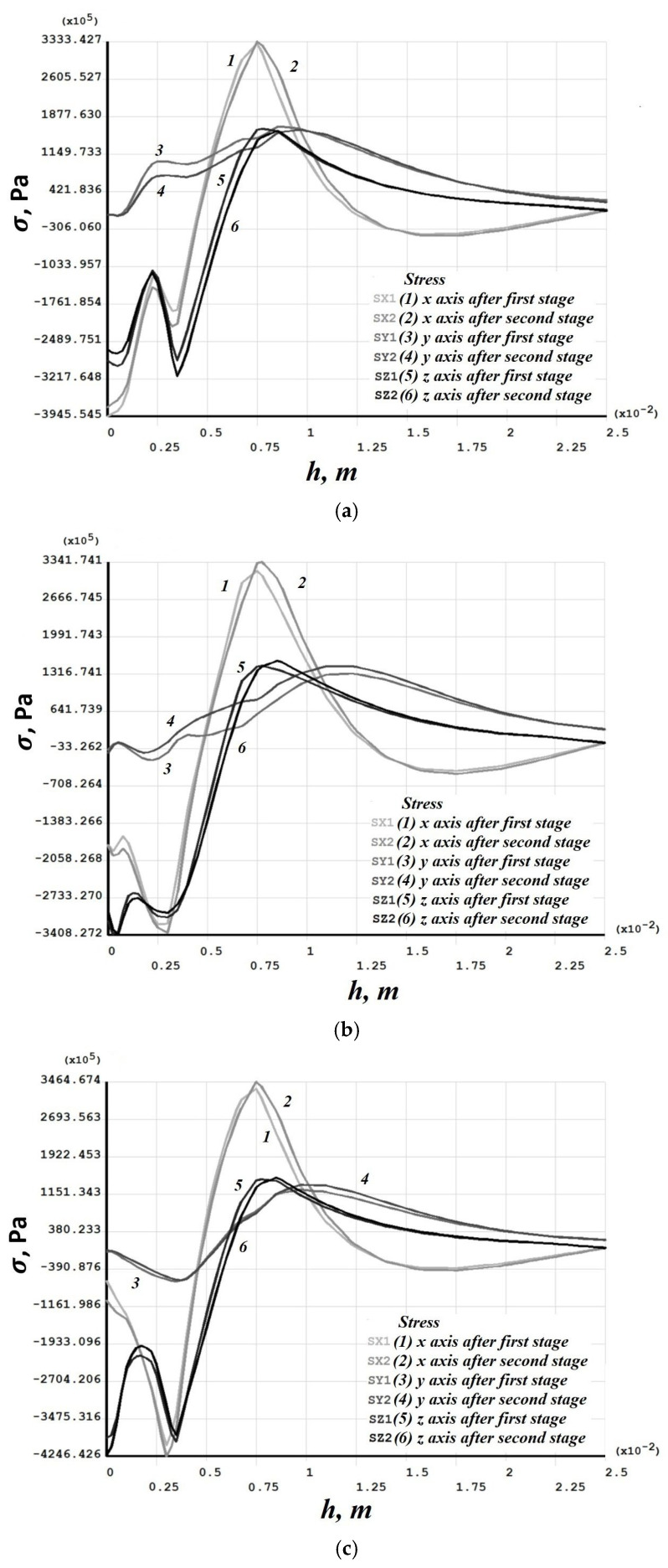
Case 2 (PC2). Distribution through the depth of the residual stress after the first and second stages: (**a**) across the height of the left (−3 mm); (**b**) central (0 mm); (**c**) right (+3 mm) sections.

**Figure 15:**
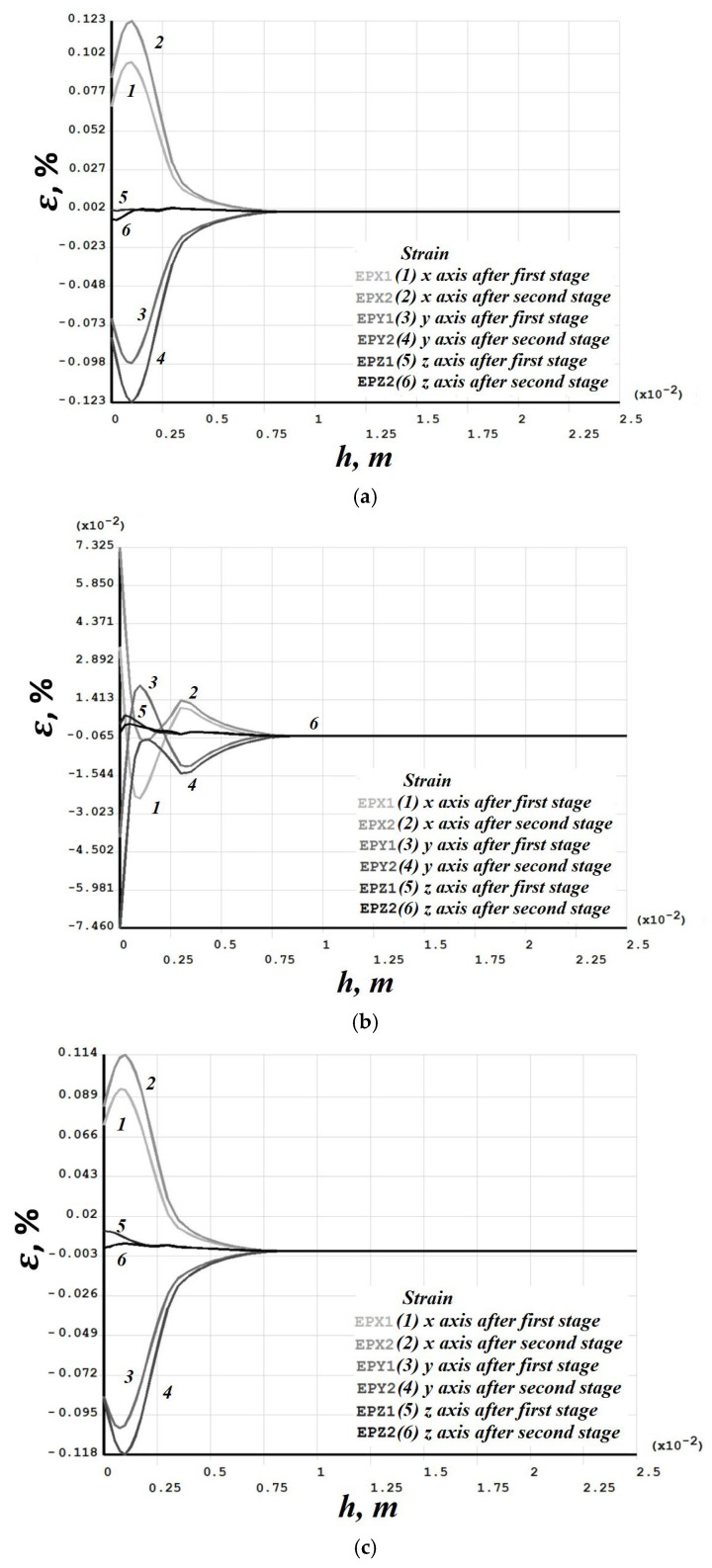
Case 2 (PC2). Distribution through the depth of the residual strain after the first and second stages: (**a**) across the height of the left (−3 mm); (**b**) central (0 mm); (**c**) right (+3 mm) sections.

**Figure 16:**
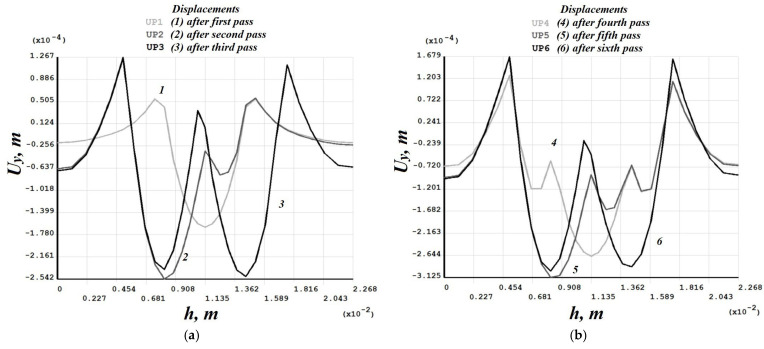
Case 2 (PC2). Distribution of vertical residual displacements along the top surface of the cross-section after the first–third (**a**) and fourth–sixth (**b**) passes.

**Figure 17:**
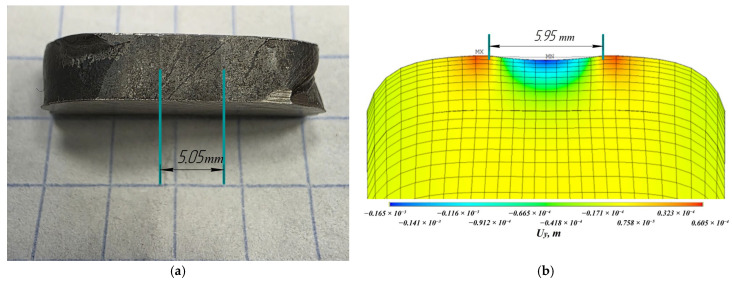
Comparison of experimental and calculated width of the central groove during single-stage forging of Inconel 718 specimen: (**a**) segment of the deposited specimen (top view); (**b**) calculated residual displacements.

Verification of the mathematical model was performed using a wall (specimen 2) deposited with single-stage forging by comparing the width and the depth of the central groove on the last layer (Figure 17). The deviation between the simulation results and the experiment did not exceed 15%, which indicates the adequacy of the proposed model. The experiment showed that the groove depth ranged from 0.14 mm to 0.15 mm, while the calculated depth was approximately 0.16 mm. The groove width, according to the experimental results, ranged from 5 to 5.1 mm, while the calculated width was approximately 5.95 mm.

Numerical simulation results (Figure 12 and Figure 16) showed that the depth of plastic deformation ranges from 2.5–2.8 mm for single-stage forging to 3.1–3.2 mm for two-stage forging at a deposited layer height of 2 mm.

These results are supported by macrostructure images (Figure 4b,c), where alternating layers of dendritic and fine-grained structures are visible. The structural refinement and thus improved mechanical properties occur owing to the recrystallisation of the deformed material during heating and cooling when the subsequent layer is deposited; this was previously described for the Ti-6Al-4V alloy [35]. The alternation of layers with different grain shapes is observed throughout the height of specimens 2 and 3. The depth of plastic deformation exceeds the melting depth during the deposition of the subsequent layer, ensuring the retention and accumulation of the forging effect throughout the deposition process. The developed mathematical model can therefore be used to determine the parameters and effectiveness of the inter-pass surface deformation during layer-by-layer deposition, in particular the depth of plastic deformation.

## 4. Conclusions

Inter-pass forging with different degrees of deformation during the WAAM of Inconel 718 (single-stage, three passes; two-stage, six passes) enables the tuning of tensile strength within 15% up to 949 MPa and the tuning of yield strength within 25% up to 624 MPa.

Two-stage forging increases the general level of microhardness and produces a more uniform distribution compared with single-stage forging. Compared with deposition without cold inter-pass surface deformation, forging increases average microhardness by 20–30 HV_0.1_.

Single-stage forging forms a fine-grained structure in the center of the specimen while the dendritic structure remains at the edges. The alternation of layers with different grain shapes is observed throughout the height of the specimens. Two-stage forging forms a recrystallized structure over almost the entire height and width of the deposited layer. The height of layers with equiaxed grains increases from 0.90–1.25 mm (single-stage) to 1.42–1.56 mm (two-stage), while the height of layers with columnar grains decreases from 0.80–1.00 mm to 0.64–0.77 mm.

A finite element model of inter-pass forging was developed to determine the effect of inter-pass surface deformation on the residual stress–strain state. Johnson–Cook material constants were obtained for the deposited Inconel 718 material, including the effect of forging. Verification of the model was performed by comparing the width and the depth of the central groove on the last layer of a wall (specimen 2) deposited with single-stage forging. The deviation between the simulation results and the experiment did not exceed 15%. The experiment showed that the groove depth ranged from 0.14 mm to 0.15 mm, while the calculated depth was approximately 0.16 mm. The groove width, according to the experimental results, ranged from 5 to 5.1 mm, while the calculated width was approximately 5.95 mm. This indicates the adequacy of the proposed model and replacing a transient formulation with a quasi-static one for Inconel 718.

Numerical modeling showed that the sequence of passes significantly affects the distribution of vertical residual displacements, though the maximum depth of the final grooves is the same. Residual stresses change little upon repeat forging (average growth ≤ 10%), whereas residual strains can increase by more than 100% in some sections after the second forging stage.

Numerical modeling showed that the depth of plastic deformation exceeds the melting depth when depositing the next layer, ensuring the preservation and accumulation of the inter-pass forging effect throughout the deposition process. The developed model can therefore be used to determine the parameters and effectiveness of inter-pass surface deformation, particularly the depth of plastic deformation, during layer-by-layer deposition.

Future experiments with other materials are planned, the results of which can be used for further verification of the mathematical model of inter-pass forging. The developed mathematical model is planned to be used in the future for the production of various metal structures to determine the parameters of the deformation effect necessary to achieve strengthening without distorting the geometry or causing structural failure. The effect of deformation effects on high-cycle fatigue characteristics will also be studied.

## Figures and Tables

**Table 1:** Chemical composition of the wire and deposited material (wt.%).

	C	Si	Mn	P	S	Cr	Mo	Ni	Cu	Ti	Al	Nb	B	Fe
Welding wire	0.06	0.05	0.02	0.0032	0.0014	19.19	2.96	52.29	0.02	0.93	0.48	5.25	0.002	18.65
Deposited material	0.06	0.05	0.02	0.0029	0.0008	19.14	2.98	51.98	0.01	0.93	0.47	5.28	0.002	18.96

**Table 2:** Dimensional characteristics of layers with different grain shapes in the macrostructure.

Layer Height	Single-Stage Forging (Specimen 2)	Two-Stage Forging (Specimen 3)
Equiaxed grain layers, mm	0.90–1.25	1.42–1.56
Columnar grain layers, mm	0.80–1.00	0.64–0.77

**Table 3:** Results of statistical analysis of microhardness (HV_0.1_).

Parameter of Microhardness (HV_0.1_)	Without Forging (Specimen 1)	Single-Stage Forging (Specimen 2)	Two-Stage Forging (Specimen 3)
Mean value	252.06	280.87	285.69
Minimum value	214.00	225.00	249.00
Maximum value	312.00	328.00	328.00
Standard deviation	17.586	16.021	15.259
Confidence interval	41.05	37.4	35.6
Range of values	211.0–293.1	243.5–318.3	250.1–321.3

**Table 4:** Results of tensile testing of Inconel 718 without subsequent heat treatment.

Material Categories	*E*, GPa	$\sigma_{B}$, MPa	$\sigma_{pl}$, MPa	$\sigma_{u}$, MPa	δ, %	ψ, %
Without forging	170 ± 10	816	350 ± 20	462	38	29
Single-stage	180 ± 15	916	450 ± 20	591	26	23
Two-stage	180 ± 15	949	460 ± 30	624	28	26

**Table 5:** Johnson–Cook constants for different categories of Inconel 718.

Source		*A*, MPa	*B*, MPa	n	C	m	${\dot{\varepsilon }}_{*}$ $,\;s^{-1}$
[46] (after heat treatment)		1290	895	0.526	0.016	1.55	0.03
From Table 4	without forging	435	650	0.526	0.016	1.55	0.03
	two-stage forging	624	520	0.526	0.016	1.55	0.03

## Data Availability

The original contributions presented in this study are included in the article. Further inquiries can be directed to the corresponding author.
